# A method for measuring the length of the coclea through magnetic resonance imaging

**DOI:** 10.1016/S1808-8694(15)30788-6

**Published:** 2015-10-19

**Authors:** Fernando Pochini Sobrinho, Paulo Roberto Lazarini, Hea Jung Yoo, Luiz de Abreu Júnior, Altino de Sá Meira

**Affiliations:** 1 Master's degree, Faculdade de Ciências Médicas da Santa Casa de São Paulo. Adjunct professor II of Otorhinolaryngology, Universidade Santo Amaro, UNISA; 2 Adjunct professor of the graduate course, Faculdade de Ciências Médicas da Santa Casa de São Paulo; 3 Radiologist, assistant professor of the Image Diagnosis Unit - Serviço de Diagnóstico por Imagem, Faculdade de Ciências Médicas da Santa Casa de São Paulo; 4 Doctorate, Faculdade de Medicina da Universidade São Paulo. Radiologist in the Hospital São Luiz; 5 Biomedic, Universidade Santo Amaro. Technical supervisor of the Serviço de Ressonância Magnética da Santa Casa de São Paulo

**Keywords:** cochlea, magnetic resonance image, three-dimensional imaging, cochlear implant

## Abstract

We know that hearing impairment affects a large part of the population. In cases of profound and bilateral hearing loss, children may have problems in speech development, as well as communication and socialization. Cochlear implants have been used as a treatment option in these cases. Today, inner ear MRI is a mandatory test in the preoperative evaluation of these individuals. In our daily routines, we wonder whether MRI can provide not only qualitative, but also quantitative data, with real cochlear linear values built from three dimension images.

**Aims:**

The aim of the present investigation is to propose a method to obtain MRI cochlear length measures from the temporal bones of cadavers.

**Material and Methods:**

We assessed three dimensional images from the cochlea of six cadavers. By overlapping digitalized rulers on these images it was possible to measure cochlear length.

**Results:**

These measures varied between 17 and 26.5 millimeters.

**Conclusions:**

We have concluded that it was possible to measure cochlear length from three dimensional MRI images, by employing the method hereby proposed.

## INTRODUCTION

Cochlear implants (House 3M®) were introduced commercially in 1972; these devices stimulate the auditory nerve directly when placed in the cochlea (tympanic ramp).^1^ As these devices are currently being use more often for the treatment of patients with hearing loss, knowledge about the anatomy of the spiral canal of cochlea - into which the electrode is placed - has become paramount.

At present, image exams are employed routinely for a diagnostic preoperative assessment of candidates for cochlear implants; the aim is to define the anatomical status of the cochlea. Computed tomography (CT) and magnetic resonance imaging (MRI) are currently used for this evaluation.^2^, ^3^, ^4^, ^5^, ^6^, ^7^, ^8^, ^9^, ^10^, ^11^, ^12^, ^13^

Jackler et al.^2^ found a 46% rate of false negative results when comparing the results of high resolution CT with surgical findings in 36 ears with implants. Nikolopoulos et al.^3^ concluded that the sensitivity rate was about 62.5% in a study of 108 children. These two papers have shown that a normal preoperative CT exam does not exclude the possibility of finding cochlear obstruction during surgery for placing a cochlear implant. This has been attributed to minor degrees of ossification and fibrosis in the cochlear canal.

In the mid-80s, MRI was introduced as a new image diagnosis tool. Felix Bloch and Edward M. Purcell - who shared the physics Nobel Prize in 1952 - developed the principles of this technique (Shampo, Kyle, 1995).^4^

Currently, MRI is the image method of choice for assessing spiral canal of cochlea obstructions.^5^, ^6^, ^7^, ^8^, ^9^, ^10^, ^11^, ^12^, ^13^, ^14^, ^15^

Casselman et al.^5^ were the first to apply CISS-3DFT MRI (constructive interference in steady state - three-dimensional Fourier transformation magnetic resonance imaging) in the study of the inner ear and the cerebellopontine angle. A study of 50 normal ears and 10 diseased ears showed detailed images of the cochlear, semicircular canals, the vestibule, and the facial and vestibulocochlear cranial nerves.

Silberman et al.^7^ assessed 40 patients with cochlear implants and commented the importance of MRI, especially in children with deep hearing loss. These authors emphasized that fibrosis is not seen in CT images; these imaging methods complement each other as preoperative evaluation tools.

Guirado et al.^6^ applied CT and MRI to study 30 patients with deep hearing loss, and found conditions such as otosclerosis, inner ear malformations, semicircular canal agenesis, the Mondini malformation, labyrinthic dysplasia, and labyrinthitis ossificans. In one of the cases, CT was normal while MRI revealed an abnormal signal and an altered cochlear configuration.

Himi et al.^13^ studied the findings of 3D reconstructed CT of the temporal bone in assessing patients for cochlear implant surgery. These authors underlined the benefits of 3D reconstructed CT images, but suggested that MRI images were superior for evaluating the patency of the perilymphatic space. They commented that CT would be more useful postoperatively, since MRI is not indicated for patients bearing cochlear implants.

Baumgartner et al.^14^ demonstrated in 30 patients that MRI could be done in patients with implants - without having to remove them - with no loss in cochlear implant function.

Arnold et al.^8^ applied CT and MRI in 10 normal hearing volunteers and 13 patients with sudden deafness, progressive hearing loss, recent tinnitus, or vertigo. This study revealed that CT was unable to satisfactorily show the membranous labyrinth, internal acoustic meatus nerves, and the cerebellopontine angle. These authors also stated that MRI with contrast (gadolinium diethylenetriaminepentaacetic acid or Gd-DTPA) increases the sensitivity of this method for detecting small tumors in the internal acoustic meatus and the cerebellopontine angle.

Gleeson et al.^15^ studied 88 cochlear implant patients retrospectively; 24 of these patients had undergone preoperative CT and MRI. Surgical findings were compared to the images. These authors concluded that the ability of both exams to predict the inner ear anatomical status was similar (79% correlation with surgical findings). In this study, associating MRI and CT did not increase the sensitivity for evaluating the patency of the spiral canal of cochlea.

Ketten et al.^16^ applied 3D reconstructed CT of the spiral canal of cochlea in 20 patients in whom a NucleusR cochlear implant was placed; the mean measured spiral canal length was 33.01 mm ± 2.31 in 3D CT, and the mean attained electrode depth was 20.19 mm ± 2.86.

Using a block inclusion technique for studying a temporal bone of a 76-year old patient for computer analysis, Takagi and Sando17 estimated the cochlear length as being 36.3 mm in a 3D calculation, as opposed to 30.8 mm in 2D graphic reconstruction techniques.

New 3D reconstructed MRI techniques have recently been described and compared. Jung et al.^18^ compared 3-dimensional driven equilibrium with sensitivity encoding and 3-dimensional balanced fast-field echo sequences and found that the 3D driven method was possibly superior for assessing inner ear structures.

Lane et al.^19^ found that both the CISS-3DFT (constructive interference in steady state - three-dimensional Fourier transformation) and the 3D FRFSE (three-dimensional fast recovery fast spin-echo) MRI techniques generated high-resolution images of the labyrinth. Naganawa et al.^20^ compared 3D rIR (fast spin-echo-based three-dimensional real inversion recovery) and 3D SPGR (three-dimensional spoiled gradient echo) T1-weighted contrasted images for visualizing small structures in the temporal bone, to find whether both techniques were useful for identifying any increased cochlear fluid volume in seven volunteers. These authors concluded that the 3D SPGR technique was superior for assessing the cochlear fluid space, while 3D rIR was better for visualizing small structures in the temporal bone. Bartling et al.^21^ proposed a method for using MRI and CT jointly to improve surgical planning for the treatment of tumors and implant placement in partially obstructed cochleae, available only for selected cases. Naganawa et al.^22^ were able to image the perilymphatic and endolymphatic (space) fluid separately at 3T, 24 hours after injecting Gd-DTPA intratympanically. These authors stated that the endolymphatic space could be differentiated from bone and air in the four patients they studied.

We asked whether MRI could provide routine qualitative and quantitative data such as true linear measurements of the cochlea in 3D reconstructed images.

The purpose of this study was to propose a technique for verifying cochlear length measurements in magnetic resonance images of cadaver temporal bones.

## MATERIAL AND METHOD

The Research Ethics Committee of the Santa Casa de Misericórdia de São Paulo approved this study on 11 September 2002 (protocol number 191/02).

Six adult cadaver temporal bones obtained from the Serviço de Verificação de Óbitos da Capital de São Paulo from 20 March 2004 to 14 April 2004 were used. These were removed with cutting tools always by the same technician within 12 hours of death and refrigerated at 4°C in an aqueous 10% formaldehyde solution (Table 1). The temporal bones originated from five female (83.33%) and one male subject (16.67%). Their mean age was 67 years.

**Table 1:** Classification of temporal bones by registration number, age and sex. Source: Serviço de Verificação de Óbitos da Capital de São Paulo.

	No. SPC	AGE (years)	GENDER
Case 1	3043	80	F
Case 2	3030	69	F
Case 3	3038	76	F
Case 4	3037	62	F
Case 5	3035	60	M
Case 6	276	55	F

The temporal bones were taken to and kept at 4°C in a 10% formaldehyde solution in the dissection room of the Otorhinolaryngology Department, Santa Casa de São Paulo, before being taken to the Diagnostic Unit of the Santa Casa de Misericórdia de São Paulo for imaging.

**Fig. 1:**
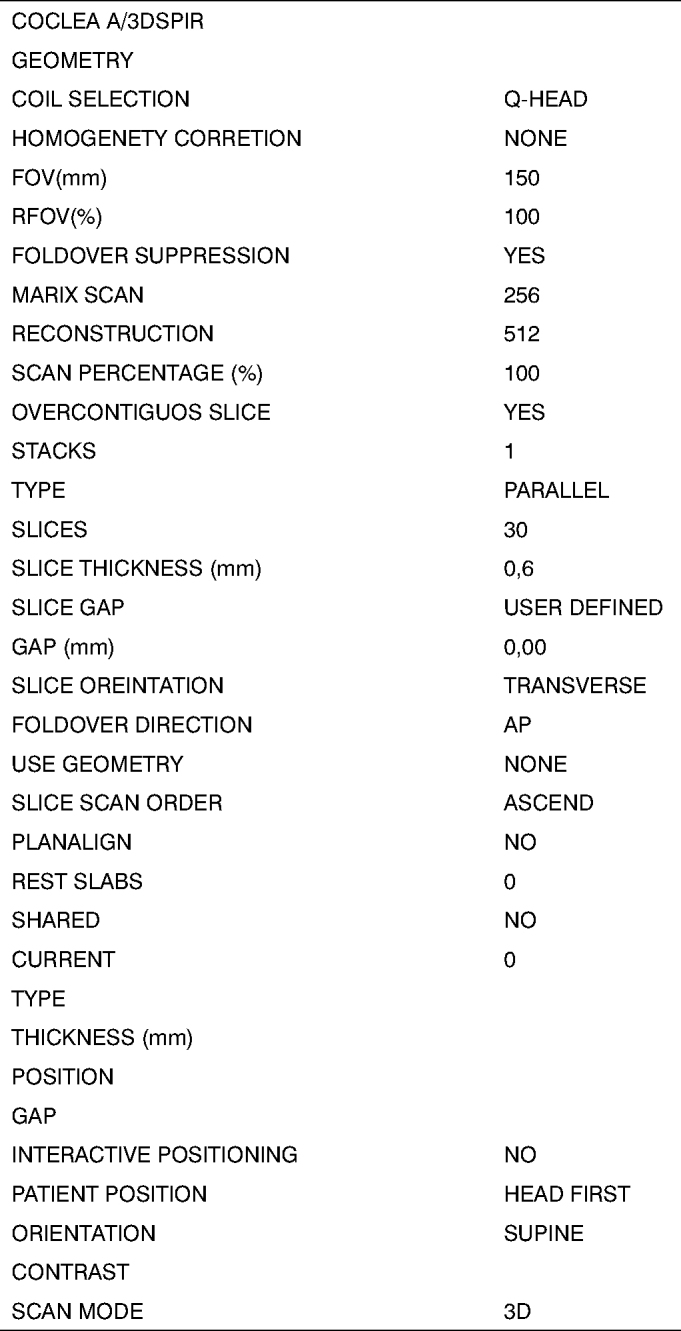
3D reconstructed MRI protocol

**Fig. 2:**
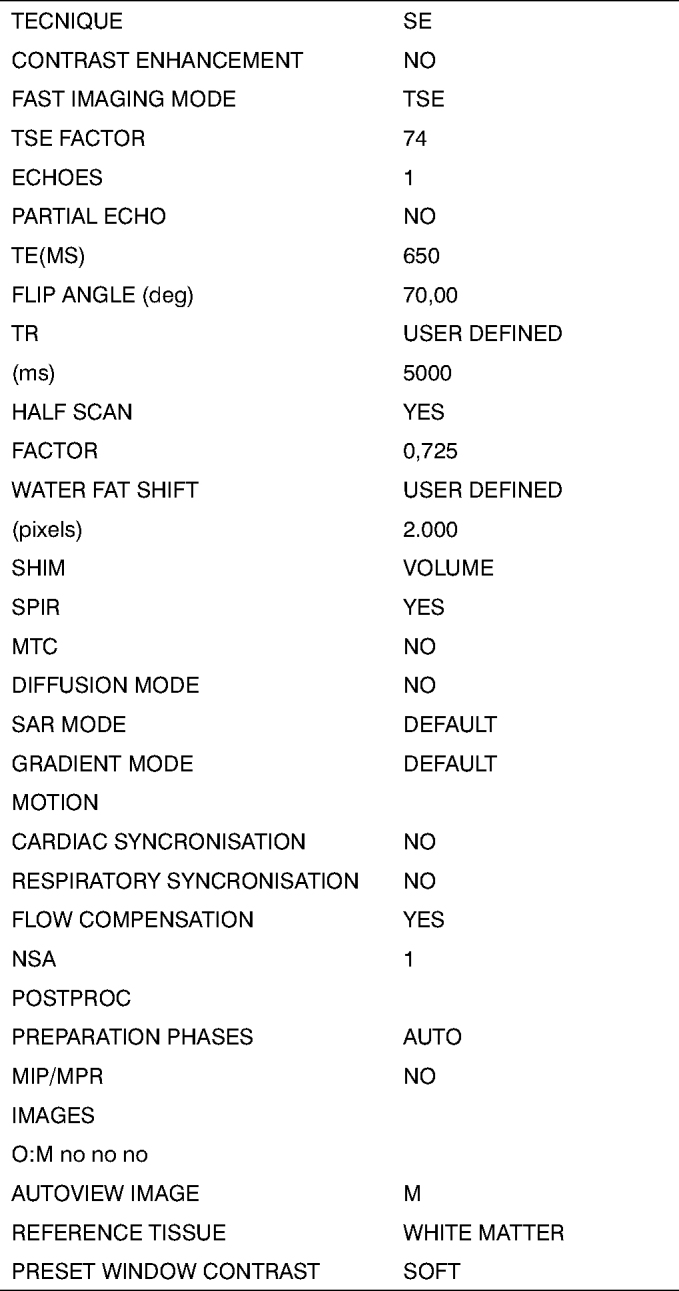
3D reconstructed MRI protocol (continued).

MRI was done within a week after the temporal bones were received. Fat-suppressed T2-weighted volume sequences (SPIR) were taken (Fig. 1, Fig. 2), from which 3D reconstructed images were obtained for cochlear measurements. A Gyroscan T10-NT Powertrak 1000 (1.0 Tesla) Philips Medical Systems magnetic resonance imaging device was used. Temporal bones were placed in a circular surface coil (C3) for the exam.

**Fig. 3:**
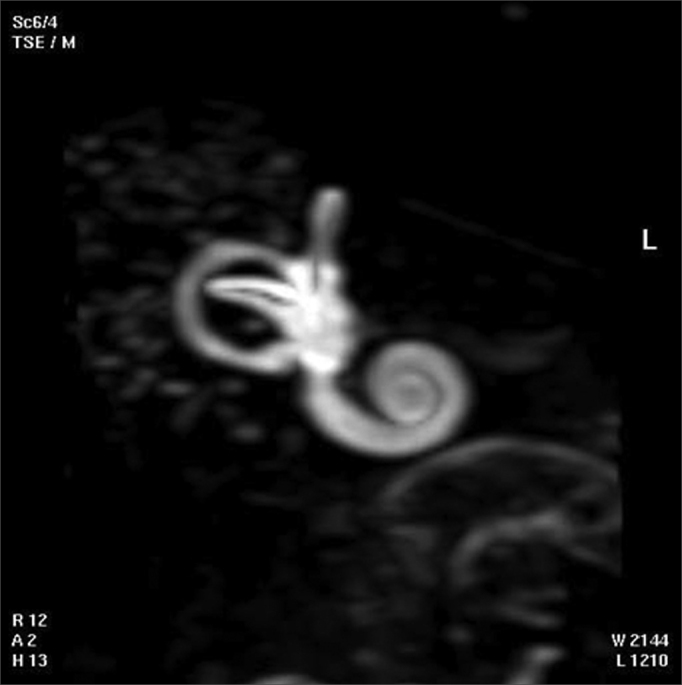
3D magnetic resonance image of case 3.

The 3D reconstructed technique was used to acquire coronal and axial images of the inner ear. The coronal section reconstruction was chosen since it increases visibility of the turns of the cochlea (Fig. 3).

Measurements were taken in six images analyzed using a computer that is part of Philips Medical Systems MRI devices. The length of the cochlea was measured first by using digitized ruler images (in millimeters), which are part of the computer software, projected onto the cochlear images. The length of the spiral canal of cochlea was measured from its closer point to the vestibule to its apex.

Given the curvature of the cochlea, it would be impossible to use this method for measuring the spiral canal of cochlea. The solution was to divide the spiral canal into a number of sections and to measure each one; adding up these values yielded the total length of the cochlea.

**Fig. 4:**
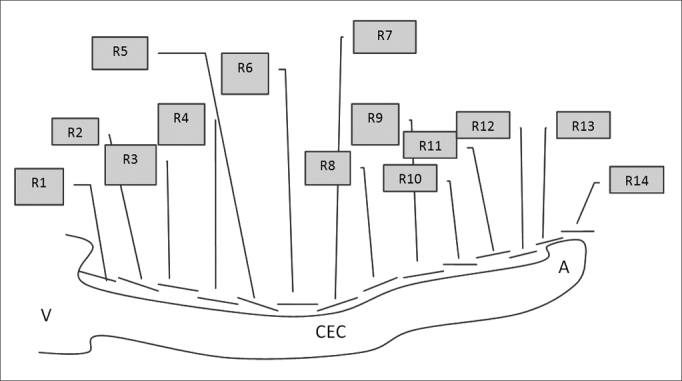
Scheme for measuring the length of the spiral canal of cochlea in 3D magnetic resonance images. R=measurement in MRI. V=vestibule, A=apex, CEC= spiral canal of cochlea.

**Fig. 5:**
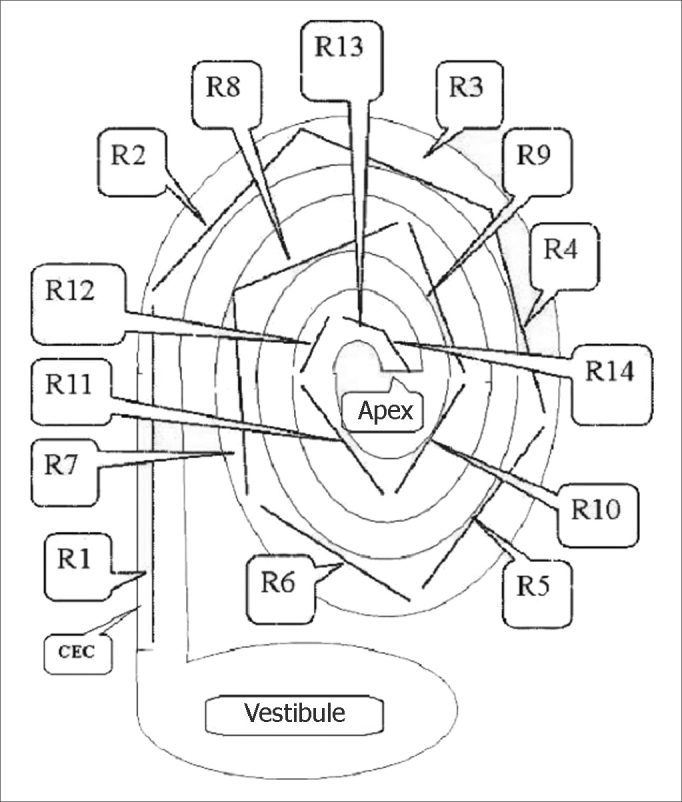
Schematic drawing showing how 3D magnetic resonance imaging measurements were done. R=measurement between two points in the spiral canal of cochlea (CEC). Measurements were done from the vestibule towards the apex of the cochlea, using 2 mm predefined measures for the first six measurements (R1, R2,……, R6) and predefined 2 mm, 1.5 mm or 1.0 mm measures for measurements from R6 to Rx (R14).

The rulers had predefined 2.0, 1.5 and 1.0 mm markings and were placed over the cochlear images for measurements. Many rulers were required for each image until reaching the apex of the cochlea. The first six rulers (R1 to R6) measured 2 mm; the next rulers had smaller sizes to measure the cochlear contours (R7 to Rx). Fig. 4, Fig. 5 show the cochlear measurement points.

## RESULTS

**Fig. 6:**
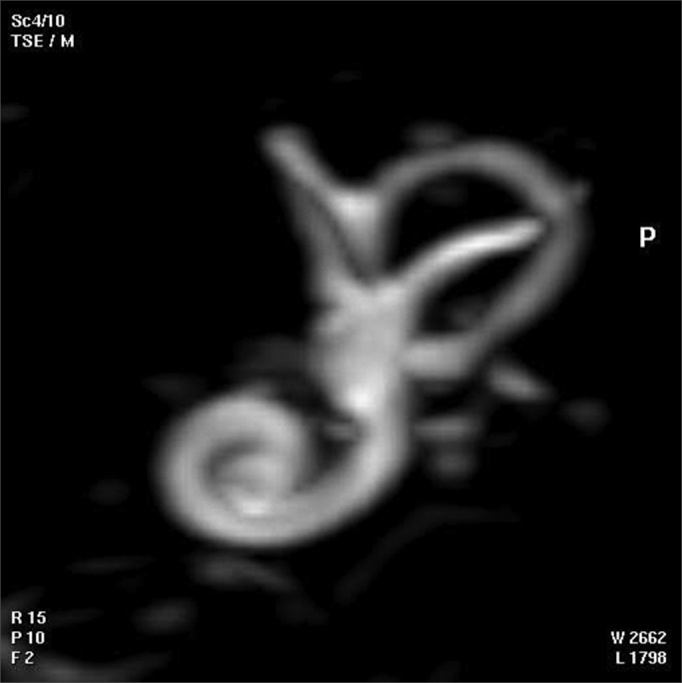
3D magnetic resonance image chosen for providing the best image of the turns of the spiral canal of cochlea in the temporal bone of case 1.

One of each 3D MRI images for cases 1 to 6 was chosen: that which provided the best view of the turns of the spiral canal of cochlea (Fig. 6, for example).

The length in 3D MRI images of the same cochlea were checked against each temporal bone (Table 2), using predefined measurements from the vestibule to the apex of the cochlea (Table 3).

**Table 2:** Measurements of the length of the spiral canal of cochlea in 3D magnetic resonance images of six human temporal bones.

	Cochlear length at the MRI (in mm)
Case 1	23,00
Case 2	19,00
Case 3	23,50
Case 4	22,00
Case 5	26,50
Case 6	17,00

**Table 3:** Values of predefined measurements - “R” (distance between two points in cochlear canal images) checked against measurements of spiral canal lengths in 3D magnetic resonance images of temporal bones. Distances are given in mm, starting at the vestibule (R1) towards the apex of the cochlea in 3D MRI images (Rx) in cases 1 to 6.

Distance	Case 1	Case 2	Case 3	Case 4	Case 5	Case 6
R1	2,00	2,00	2,00	2,00	2,00	2,00
R2	2,00	2,00	2,00	2,00	2,00	2,00
R3	2,00	2,00	2,00	2,00	2,00	2,00
R4	2,00	2,00	2,00	2,00	2,00	2,00
R5	2,00	2,00	2,00	2,00	2,00	2,00
R6	2,00	2,00	2,00	2,00	2,00	2,00
R7	2,00	2,00	2,00	2,00	2,00	1,50
R8	2,00	2,00	2,00	2,00	2,00	1,50
R9	2,00	1,00	2,00	2,00	2,00	1,00
R10	1,50	1,00	1,50	1,50	2,00	1,00
R11	1,50	1,00	1,50	1,50	2,00	DNM
R12	1,00	DNM	1,50	1,00	2,00	DNM
R13	1,00	DNM	1,00	DNM	1,50	DNM
R14	DNM	DNM	DNM	DNM	1,00	DNM
*length (mm)*	23,00	19,00	23,50	22,00	26,50	17,00

The results among the six cochleae may be compared in Table 3.

## DISCUSSION

Otological surgery of the inner ear has become more frequent, which requires surgeons to have a more detailed knowledge of the anatomy, measurements and landmarks of the labyrinth. This is needed for developing new surgical techniques and for placing devices such as implants, valves and others. Cochlear implants placed on the tympanic ramp (House, 1972)^1^ have led to an increased number of procedures in the inner ear.

CT and MRI are the imaging methods used for assessing the spiral canal of cochlea. According to Gleeson et al.^16^ there were no significant differences between CT and MRI in the preoperative assessment for cochlear implants; no benefit has been observed by using both methods together. Himi et al.^15^ have stated that 3D MRI is superior compared to CT for assessing the patency of the perilymphatic space. Jackler et al.^2^ and Nikolopoulos et al.^3^ have stated that a normal preoperative CT does not exclude the possibility of finding an obstructed cochlea during surgery. MRI has thus been used as the imaging method of choice for assessing spiral canal obstructions, especially in candidates for cochlear implants.^4^, ^5^, ^6^, ^7^, ^8^, ^9^, ^10^, ^11^, ^12^, ^13^, ^14^

Three-dimensional MRI provided high quality images of the spiral canal of cochlea in our cadaver temporal bone cases.

Takagi and Sando (1989)^18^ compared a 3D computerized cochlear measurement method with 2D measurements of temporal bone histological sections, and found that the mean cochlear length was 36.3 mm in 3D measurements and 30.28 mm in 2D measurements. In our cases the computer may have altered the true measurements of the cochlea, compared to direct measurement methods. Three-dimensional computer technique measurements of histological sections by Takagi and Sando^18^ yielded higher values than those found in our six cases.

Ketten et al.^17^ measured the spiral canal of cochlea preoperatively using CT and during cochlear implant surgery, and found that the mean length of the cochlea was about 33.01 mm (standard deviation – 2.31 mm). These values are far from those in our 3D MRI images (17 to 26.50 mm).

Our 3D MRI image measurements were not similar to the values measured using CT in the abovementioned studies, which appear to provide measurements that are closer to the true size of the cochlea. In our cases, the size of the cochlea in the images was smaller. Additionally, measurements of cases 2 and 6 were much smaller compared to the remaining cases. This may have been due to an underestimated MRI measurement or true variability in the size of the cochlea in the temporal bones we studied.

New 3D reconstructed MRI techniques have been described recently.^18^, ^19^, ^20^, ^21^, ^22^

A study of a larger series and improved image acquisition techniques may yield more precise cochlear measurements, which may then be used in routinely in otorhinolaryngology. This may provide the means for using MRI to accurately measure the size of the cochlea in implant patients. Procedures may then be tailored individually, varying the size and distribution of electrodes within the cochlea, thus providing improved stimulation across the cochlea.

## CONCLUSION

The length of the cochlea in cadaver temporal bones was measured with a technique that uses rulers over 3D reconstructed MRI images of those bones. The results appear to be shorter than those described in the literature.
